# Impact of air pollution and behavioral factors on cognitive decline among middle-aged and elderly populations in China: a retrospective cohort study based on CHARLS

**DOI:** 10.1186/s12889-025-23211-3

**Published:** 2025-07-02

**Authors:** Zhi Yu, Liqiang Su, Pinshi Ni, Jiahan He, Yingmin Su, Jianmei Cui, Fanghui Li

**Affiliations:** 1 School of Sport Sciences, Nanjing Normal University, Nanjing, China; 2 Jiangxi Normal University, Nanchang, China; 3 North University of China, Taiyuan, China

**Keywords:** Cognitive, Cognitive decline, Air pollution, Middle-aged and elderly population

## Abstract

This retrospective cohort study was based on the China Health and Retirement Longitudinal Study (CHARLS) database, utilizing longitudinal data collected from 2011 to 2018 and comprising 65,159 person–wave observations from 23,412 unique participants, combined with a high-resolution air pollution model, to systematically assess the impact of multidimensional factors (sociodemographic characteristics, lifestyle, health status, and air pollution) on cognitive decline in middle-aged and elderly individuals. The study revealed that age is the primary factor affecting cognitive function, which decreases with increasing age. Through stratified regression and interaction tests, factors significantly affecting cognitive decline were identified and ranked according to importance via a random forest model. The results indicated that PM_2.5_ was the most significant factor affecting cognitive decline, regardless of sex, followed by fuel type usage, BMI, and physical activity, among other factors. This study comprehensively integrated the effects of air pollution and multidimensional factors on cognitive decline, providing a scientific basis for developing gender-specific and personalized cognitive intervention strategies.

**Supplementary Information:**

The online version contains supplementary material available at 10.1186/s12889-025-23211-3.

## Introduction

Cognitive decline is a progressive disease associated with aging that typically involves deterioration of memory, judgment, and the ability to learn new skills [1]. According to the United Nations, the global population aged 65 and older is expected to increase from 700 million in 2019 to 1.6 billion by 2050 [2]. China is one of the countries most affected by population aging; by the end of 2023, the number of people aged 60 and over had reached 296.97 million, accounting for 21.1% of the total population. The consequences of cognitive decline are significant; it can lead to a decreased ability to live independently, reduced social participation, deteriorated mental health, and even an increased risk of death [3].

Research has confirmed that changes in cognitive function are influenced by a variety of factors, including sociodemographic characteristics, lifestyle, health status, and environmental exposure [4, 5]. For example, sociodemographic factors such as educational level, occupation type, and socioeconomic status are widely considered to be closely related to cognitive function [6]; a healthy lifestyle, including regular physical exercise, a healthy diet, and good sleep quality, helps maintain cognitive function [7]; and chronic diseases such as diabetes, hypertension, and obesity have been shown to be related to cognitive decline [8, 9]. Additionally, air pollutants, particularly PM_2.5_ and nitrogen oxides (NO_x_), are attracting increasing attention because of their potential impact on brain health [10].

However, current research still has several shortcomings. First, most studies on cognitive function are focused on developed countries, with relatively few studies in developing countries, especially in areas such as China, which has unique social and environmental backgrounds. Second, due to the lack of high-quality data on long-term systematic monitoring of air pollutants in China, epidemiological evidence on the impact of air pollution on cognitive function is scarce [11]. Third, existing studies often focus on single-dimensional factors, such as sociodemographic characteristics or health status, and lack a systematic exploration of the interactions and comprehensive effects of multidimensional factors [12, 13].

Therefore, we attempt to use nationally representative longitudinal survey data—the China Health and Retirement Longitudinal Study (CHARLS)—combined with high-resolution air pollution satellite models to systematically assess the impact of multidimensional factors on cognitive function in middle-aged and elderly individuals. This approach aims to provide data support for developing gender-specific and regional health intervention policies, help optimize cognitive health management strategies, and offer theoretical and practical guidance for addressing the challenges of aging and environmental health in China.

## Study subjects and methods

### Study population and cohort design

The study subjects were derived from the China Health and Retirement Longitudinal Study (CHARLS), an ongoing nationally representative longitudinal study initiated by Peking University. It aims to collect high-quality microdata representing families and individuals aged 45 and over in China to analyze the aging population issue and promote interdisciplinary research on aging [14]. CHARLS uses stratified, multistage random sampling, covering 126 cities, 150 county-level units, and 450 village-level units across the country, involving 17,708 individuals from 10,257 households. The baseline survey was conducted in 2011, followed by bi to triennial follow-ups via structured questionnaires and physical examinations in face‒to-face interviews to collect information on sociodemographic, lifestyle, and health-related factors (http://charls.pku.edu.cn/). The survey participants were randomly selected residents aged 45 years and above from the sampled households. Each survey round utilized computer-assisted interviewing technology, with trained interviewers collecting data on respondents’ socioeconomic and demographic characteristics, lifestyle, behavioral patterns, and health status via standardized questionnaires. The CHARLS project has been reviewed and approved by the Biomedical Ethics Review Committee of Peking University (Human Metrics Review Approval No. IRB00001052-11015; Biomarkers Review Approval No. IRB00001052-11014), and all participants signed informed consent forms, Prior to each survey wave, uniformly trained interviewers visited participants in their homes (or at community activity centers) to provide a detailed oral explanation of the study’s objectives, survey content, data confidentiality measures, and participants’ rights, and to distribute a written informed consent form. Only after participants had fully understood the information and returned a signed consent form did the formal interview proceed. The baseline wave (2011) and the follow-up waves in 2013, 2015, and 2018 all adhered to this same procedure. This study used baseline (CHARLS-2011) and three subsequent follow-up (CHARLS-2013, CHARLS-2015, CHARLS-2018) datasets, creating longitudinal data linked by unique baseline population identifiers. Health status and related information were confirmed by the respondents themselves or their family members during face‒to-face interviews. The inclusion and exclusion criteria for the study subjects were as follows: the 2011 national baseline survey included 17,705 participants, with follow-ups in 2013 surveying 18,634 people, 21,088 in 2015, and 19,816 in 2018. In this study, the exclusion criteria were as follows: (1) 2,030 participants under the age of 45 years, (2) 5,824 participants with missing cognitive function scores, (3) 3,024 participants with brain damage or intellectual deficits, and (4) 1,206 participants with memory disorders. Consequently, the sample comprised 14,975 participants in 2011 (Wave 1), 15,240 in 2013 (Wave 2), 17,043 in 2015 (Wave 3), and 17,901 in 2018 (Wave 4), resulting in 65,159 person-wave observations from 23,412 unique individuals.

### Observation indicators

In the CHARLS, sociodemographic information includes age, sex (male, female), and marital status (with spouse, without spouse); lifestyle information includes smoking and drinking habits, physical activity (low intensity/no exercise and medium-high intensity), and sleep duration (< 6 h and ≥ 6 h), with the use of biofuels for heating and cooking representing indoor air pollution; and health information includes BMI and self-reported chronic diseases (yes, no).

Physical activity levels are defined on the basis of the calculation methods and rules given in the International Physical Activity Questionnaire (IPAQ), with the following formula: weekly physical activity = metabolic equivalent (MET) × frequency per week (days/week) × duration per day (minutes/day) [15]. During the CHARLS survey, participants were asked about the days and duration per week they engaged in high-, medium-, and low-intensity physical activities. The MET values for high, medium, and low intensities are 8, 4, and 3.3, respectively. A weekly physical activity ≥ 600 MET-minutes is defined as low intensity, 600–3000 MET-minutes as medium intensity, and > 3000 MET-minutes as high intensity. Given the Chinese physical activity guidelines, which recommend moderate to vigorous exercise for elderly health, medium- and high-intensity exercise are combined into medium- to high-intensity exercise, classifying physical activity into low- and medium- to high-intensity exercise. BMI was categorized according to the World Health Organization (WHO) standards: BMI < 18.5 was considered underweight, 18.5 ≤ BMI < 24.9 was considered normal weight, and BMI ≥ 25 was considered overweight or obese.

Additionally, we focus on air pollution. Residential addresses collected from the questionnaire are converted to specific cities, which are then matched with the annual exposure concentrations of air pollutants (PM_2.5_, PM _10_, NO_2_, SO_2_, O_3_, and CO), allowing for an assessment of each resident’s exposure at the city level during the corresponding survey period. In the main model, the exposure concentration from the year prior to each follow-up time point is used as the exposure variable for analysis. Annual average concentration data for air pollutants are sourced from the China Air Pollution (CHAP) dataset [16–20]. This dataset, which uses big data and artificial intelligence technologies, produces high-resolution atmospheric pollution data with a spatial resolution of 1 km × 1 km, except for nitrogen dioxide and carbon monoxide, which are at a 10 km × 10 km resolution (Fig S1). Briefly, the dataset estimates concentrations of air pollutants by integrating extensive big data from ground observations, satellite remote sensing products, emission inventories, and atmospheric reanalysis via spatiotemporally random forest models. The model parameters, validated via tenfold cross-validation, demonstrate high accuracy and predictive capability, indicating that the estimated air pollutants in this study closely align with ground measurement results (Table S1).

### Assessment of cognitive function

The CHARLS assesses the cognitive function of respondents via two composite measurement methods: mental status and episodic memory. The assessment of mental status includes the Telephone Interview for Cognitive Status (TICS) and a drawing test. The TICS consists of 10 questions where respondents must answer the date, day of the week, or current season and perform serial subtraction of 7 from 100. The number of correct answers constitutes the TICS score (maximum of 10 points). The drawing test, which assesses visual and spatial abilities, requires respondents to copy two overlapping pentagons; successful copying earns 1 point. The total mental status score is the sum of the scores from the TICS and the drawing test (maximum of 11 points). Episodic memory is measured through immediate and delayed recall tests. The interviewer reads out 10 Chinese nouns in succession, which the respondents must repeat as many as possible and recall again after 5 min. The final score for episodic memory is the average of the number of correctly recalled words from both the immediate and delayed recall tests (maximum of 10 points). The combined scores from these two measurement methods indicate the cognitive function of the respondents; higher scores suggest better cognitive function.

### Statistical analysis

Data processing and statistical analysis were conducted via R software version 4.4.2. Random forest imputation was used to address missing data. Except for body mass index (BMI), which had a missing rate of 29.54%, all other sociodemographic, lifestyle, and health covariates exhibited missing rates between 0% and 5.37%. Assuming data were missing at random (MAR), we imputed all covariates with missing values using the missForest package. When the maximum number of iterations (maxiter) was set to eight, the average out-of-bag (OOB) error stabilized (Fig S2); thus, we specified ntree = 100 and maxiter = 8 for the imputation, with all other parameters left at their default values. The resulting complete dataset was used for the primary analysis.

Sociodemographic, lifestyle, and health data are presented as percentages (%), whereas age, cognitive function, and air pollution data are expressed as the means ± standard deviations (x̅±s). The regression analysis results are presented with regression coefficients β (95% confidence interval) and P values. Prior to fitting the logistic regression models, multicollinearity among all candidate predictors was assessed by calculating variance inflation factors (VIFs). All variables exhibited VIFs below 5 (Table S2), indicating no evidence of serious multicollinearity; therefore, no predictors were removed or combined.The chi-square test describes the characteristics of categorical variables such as sociodemographics, lifestyle, and health among the study population. Because the continuous variables did not meet the assumptions of normality or homogeneity of variance (Table S3), nonparametric Mann–Whitney U tests were employed to summarize continuous characteristics of the study population—namely, age, cognitive function scores, and exposure to air pollutants. The random forest model identified key variables, moderating variables, and irrelevant variables affecting cognitive function. Univariate analysis was used to test the effect of age on cognitive function. The logistic regression model was used to test the interaction effects of moderating variables and key variables on cognitive function. The random forest model ranked the importance of variables significantly affecting cognitive decline. In the regression analysis and interaction tests, air pollutants were dichotomized according to the World Health Organization’s (WHO) “Global Air Quality Guidelines” and China’s “Ambient Air Quality Standards” (GB 3095–2012). High PM_2.5_ > 35 µg/m^3^, low PM_2.5_ ≤ 35 µg/m^3^; high PM_10_ > 70 µg/m^3^, low PM_10_ ≤ 70 µg/m^3^; high NO_2_ > 40 µg/m^3^, low NO_2_ ≤ 40 µg/m^3^; high SO_2_ > 60 µg/m^3^, low SO_2_ ≤ 60 µg/m^3^; high O_3_ > 160 µg/m^3^, low O_3_ ≤ 160 µg/m^3^. For CO, whose concentration limits are set at 4 mg/m^3^ by both guidelines and standards and with all cities in China being within this limit, the median was used to dichotomize CO into high CO > 1.7 mg/m^3^ and low CO ≤ 1.17 mg/m^3^. All the statistical tests were two-sided, with *P* < 0.05 considered statistically significant.

## Results

### Demographic characteristics of the study population (basic information)

Table 1 shows that the study included 65,159 person-wave observations, comprising 31,577 male and 33,582 female observations. The average age of the men was 60.01 ± 9.54 years, and that of the women was 59.09 ± 9.62 years. The chi-square test indicated that, except for exercise habits and fuel usage, differences in other indicators between men and women were statistically significant (*P* < 0.05). The Mann‒Whitney U test revealed that the differences in age and cognitive function between men and women were statistically significant (*P* < 0.05), whereas the differences in exposure to air pollutants were not statistically significant (*P* > 0.05).

**Table 1:** Demographic characteristics of the study population (chi-square test) according to the Mann‒Whitney U test

Indicator	Female (*N* = 31577)	Male (*N* = 33582)	X^2^/U	*p*
Age	60.01 ± 9.54	59.09 ± 9.62	295,619,273	< 0.001
Cognitive	11.47 ± 3.84	9.81 ± 4.47	217,400,942	< 0.001
PM_2.5_	63.28 ± 19.63	63.25 ± 19.68	279,064,741	0.786
PM_10_	109.13 ± 35.27	109.21 ± 35.42	278,695,584	0.982
NO_2_	35.71 ± 10.83	35.77 ± 10.91	275,708,409	0.686
SO_2_	36.07 ± 18.48	36.11 ± 18.50	129,079,146	0.832
O_3_	81.76 ± 10.45	81.85 ± 10.31	277,022,482	0.268
CO	1.25 ± 0.44	1.24 ± 0.43	130,432,262	0.674
Smoking	61.16%	3.98%	14,779.58	< 0.001
Drinking	57.49%	14.02%	9,812.85	< 0.001
Chronic disease	64.46%	68.22%	69.74	< 0.001
Marital status			465.28	< 0.001
Married	91.11%	84.58%		
Unmarried	8.89%	15.42%		
Physical activity			0.10	0.76
Low intensity/no exercise	66.17%	66.31%		
Moderate to high intensity	33.83%	33.69%		
Sleep			110.08	< 0.001
Less than 6 h	49.41%	54.24%		
More than 6 h	50.59%	45.76%		
Fuel Type			3.21	0.073
Solid	47.71%	46.73%		
Clean	52.29%	53.27%		
BMI	39.16%	50.84%	518.59	< 0.001
Underweight	6.28%	5.50%		
Normal weight	54.56%	43.66%		
Overweight/obese	39.16%	50.84%		

### Identifying key and moderating variables affecting cognitive function

**Fig. 1:**
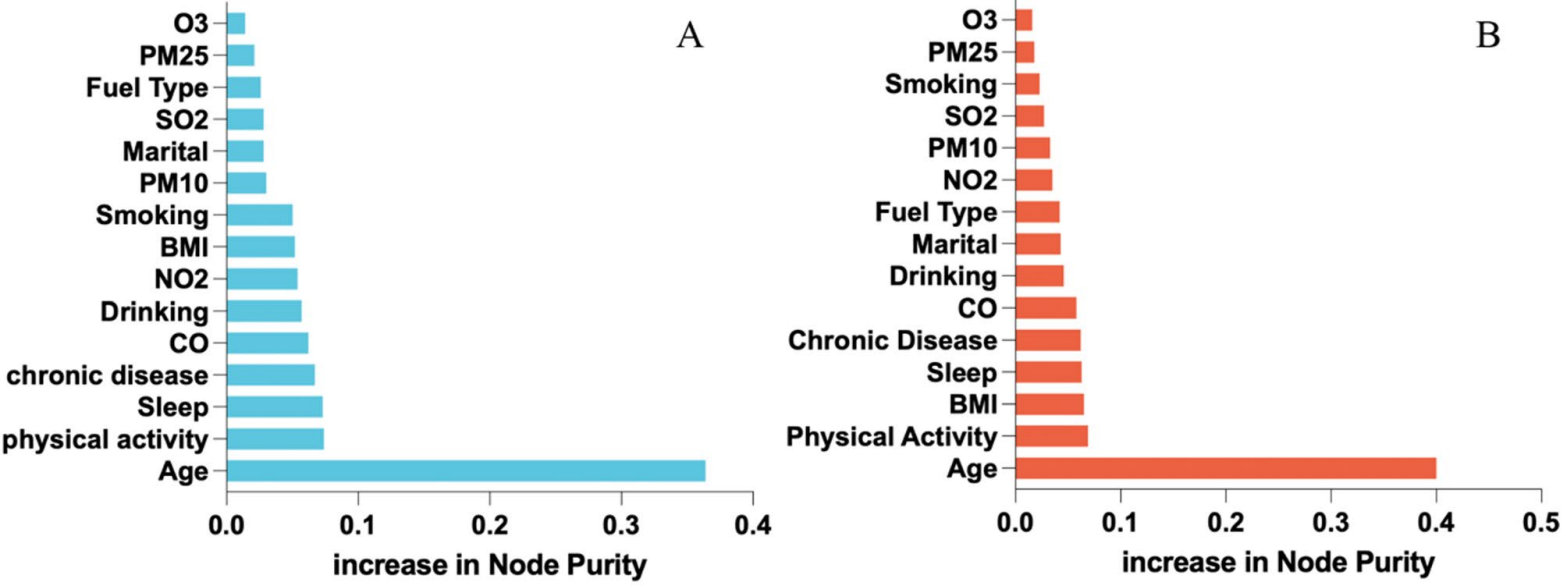
Ranking of variables potentially affecting cognitive function in males (**A**) and females (**B**)

To identify the main factors affecting cognitive function, this study uses a random forest model to rank the importance of candidate variables, uses the IncNodePurity metric as the measure, and employs the Boruta algorithm to test the statistical significance of each variable. According to the Boruta algorithm, variables are categorized as key variables, moderating variables, or irrelevant variables, with irrelevant variables excluded from further analysis. During the model fitting process, the complete sample underwent 2,000 iterations divided between the training set (40%) and the validation set (60%). The analysis shows high consistency in the categorization of variables between the male and female groups. Age has been identified as a key variable with a decisive impact on cognitive function, ranking highly in importance in the random forest model, and its statistical significance was confirmed by the Boruta algorithm. Most variables, such as BMI and PM_2.5_ concentration, are classified as moderating variables. Although they have a significant impact on cognitive function, their importance ranking and effect strength are relatively low. Additionally, O_3_ is identified as an irrelevant variable in both groups, ranking low in importance in the random forest and found not to be significant by the Boruta algorithm (Fig. 1).

### Relationships between age, environmental, and behavioral factors and cognitive function

Regression analysis was conducted with age as the independent variable and cognitive ability as the dependent variable. Table 2 show that age is an independent risk factor for cognitive decline; in the unadjusted model, for each additional year of age, cognitive ability decreased by 0.119 points, a statistically significant difference (*p* < 0.05). After adjusting for moderating variables such as BMI, type of fuel, sleep duration, and chronic diseases in the regression model, the negative effect coefficient of age on cognitive function did not change substantially. This further confirms that age is a key variable in changes in cognitive function, whereas other variables primarily act as moderators.

**Table 2:** Regression analysis of the impact of age on cognitive function

	Female β(95%CI) *p*	Male β(95%CI) *p*
Main model	-0.119(-0.124, -0.114) < 0.001	-0.166(-0.172, -0.161) < 0.001
Adjusted for BMI	-0.110(-0.115, -0.105) < 0.001	-0.162(-0.167, -0.156) < 0.001
Adjusted for fuel type	-0.117(-0.122, -0.112) < 0.001	-0.160(-0.166, -0.155) < 0.001
Adjusted for sleep	-0.119(-0.124, -0.114) < 0.001	-0.165(-0.170, -0.159) < 0.001
Adjusted for chronic diseases	-0.120(-0.125, -0.115) < 0.001	-0.164(-0.169, -0.158) < 0.001
Adjusted for physical activity	-0.119(-0.124, -0.114) < 0.001	-0.166(-0.172, -0.161) < 0.001
Adjusted for smoking	-0.121(-0.126, -0.116) < 0.001	-0.167(-0.172, -0.161) < 0.001
Adjusted for drinking	-0.116(-0.121, -0.111) < 0.001	-0.166(-0.172, -0.161) < 0.001
Adjusted for CO	-0.119(-0.124, -0.114) < 0.001	-0.165(-0.170, -0.159) < 0.001
Adjusted for NO_2_	-0.119(-0.124, -0.114) < 0.001	-0.165(-0.170, -0.159) < 0.001
Adjusted for marital status	-0.111(-0.116, -0.106) < 0.001	-0.167(-0.172, -0.161) < 0.001
Adjusted for PM_10_	-0.120(-0.125, -0.115) < 0.001	-0.165(-0.170, -0.159) < 0.001
Adjusted for SO_2_	-0.119(-0.124, -0.114) < 0.001	-0.165(-0.170, -0.159) < 0.001
Adjusted for PM_2.5_	-0.121(-0.126, -0.116) < 0.001	-0.165(-0.170, -0.159) < 0.001

### The impact of moderating variables on cognitive decline

A univariate analysis using age as the independent variable and cognitive function as the dependent variable showed that age was an independent risk factor for cognitive performance; accordingly, the age-related decline in cognitive function was defined as “cognitive decline.” To investigate the influence of other potential moderators on cognitive decline, interaction terms of each moderator variable × age were introduced. The results from Table 3 indicate that for men, BMI, fuel usage, physical activity, PM_10_, and PM_2.5_ are significant factors influencing cognitive decline (*p* < 0.05). For women, BMI, fuel usage, physical activity, drinking, marital status, PM_10_, and PM_2.5_ significantly affected cognitive decline (*p* < 0.05).

**Table 3:** Stratified regression and interaction tests

	Female		Male	
	β (95%CI)	Interaction	β (95%CI)	Interaction
BMI		0.001		0.001
Underweight	-0.121(-0.135, -0.108)		-0.166(-0.171, -0.161)	
Normal weight	-0.111(-0.125, -0.098)		-0.154(-0.168, -0.141)	
Overweight/obese	-0.091(-0.104, -0.078)		-0.138(-0.160, -0.125)	
Fuel Type		< 0.001		< 0.001
Solid	-0.120(-0.130, -0.110)		-0.168(-0.173, -0.163)	
Clean	-0.089(-0.102, -0.076)		-0.137(-0.161, -0.114)	
Sleep		0.453		0.296
Less than 6 h	-0.115(-0.128, -0.103)		-0.158(-0.171, -0.146)	
More than 6 h	-0.118(-0.125, -0.111)		-0.162(-0.168, -0.156)	
Chronic disease		0.097		0.112
Yes	-0.113(-0.125, -0.100)		-0.155(-0.169, -0.142)	
No	-0.123(-0.130, -0.117)		-0.165(-0.171, -0.160)	
Physical activity		< 0.001		< 0.001
Low intensity/no exercise	-0.140(-0.152, -0.129)		-0.188(-0.202, -0.175)	
Moderate to high intensity	-0.106(-0.112, -0.099)		-0.155(-0.161, -0.149)	
Smoking		0.137		0.408
Yes	-0.115(-0.128, -0.102)		-0.155(-0.171, -0.141)	
No	-0.123(-0.130, -0.117)		-0.166(-0.172, -0.161)	
Drinking		0.480		0.001
Yes	-0.115(-0.128, -0.103)		-0.189(-0.211, -0.167)	
No	-0.118(-0.126, -0.111)		-0.162(-0.168, -0.156)	
CO		0.294		0.602
High	-0.115(-0.128, -0.103)		-0.168(-0.180, -0.156)	
Low	-0.121(-0.127, -0.115)		-0.165(-0.172, -0.159)	
NO_2_		0.384		0.281
High	-0.115(-0.128, -0.103)		-0.161(-0.175, -0.148)	
Low	-0.120(-0.126, -0.114)		-0.168(-0.175, -0.162)	
Marital status		0.730		0.002
Married	-0.112(-0.117, -0.106)		-0.155(-0.162, -0.149)	
Unmarried	-0.111(-0.124, -0.098)		-0.178(-0.192, -0.164)	
PM_10_		< 0.001		< 0.001
High	-0.123(-0.137, -0.110)		-0.173(-0.191, -0.155)	
Low	-0.096(-0.110, -0.083)		-0.135(-0.150, -0.121)	
SO_2_		0.934		0.556
High	-0.118(-0.132, -0.104)		-0.172(-0.192, -0.152)	
Low	-0.119(-0.124, -0.114)		-0.166(-0.171, -0.160)	
PM_2.5_		0.020		0.001
High	-0.122(-0.136, -0.108)		-0.170(-0.190, -0.150)	
Low	-0.098(-0.118, -0.078)		-0.133(-0.155, -0.110)	

### Ranking of the importance of factors affecting cognitive decline

**Fig. 2:**
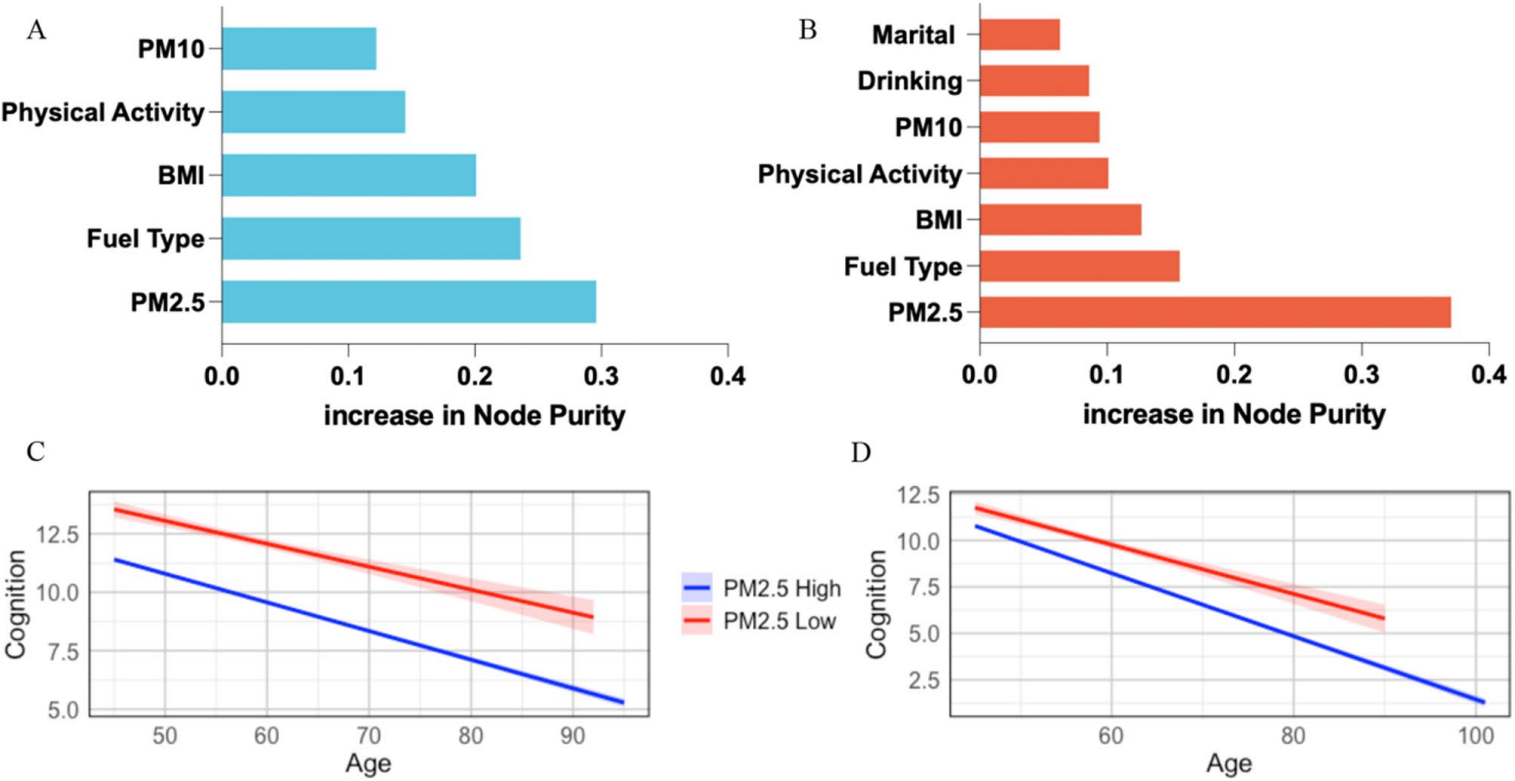
Ranking of the importance of factors affecting cognitive decline in males (**A**) and females (**B**) and the impact of PM_2.5_ on cognitive decline in males (**C**) and females (**D**)

Through stratified regression and interaction tests, we identified factors significantly affecting cognitive decline across different sex groups. These factors were ranked by importance via a random forest model. During model fitting, the complete sample underwent 5000 iterations across the training set (40%) and validation set (60%). Figure 2 shows the importance rankings of these significant factors in the random forest model, with the results expressed as percentages. These factors are highly consistent in their impact on cognitive decline across genders. PM_2.5_ is identified as the most crucial factor affecting cognitive decline, regardless of sex. The subsequent factors include fuel type, BMI, physical activity, and PM_10_. Factors unique to females, such as alcohol consumption and marital status, rank last.

## Discussion

This study utilized nationally representative CHARLS data to systematically assess the impact of environmental and behavioral factors on cognitive decline in middle-aged and elderly individuals. These results indicate that age is an independent risk factor for cognitive decline. Air pollutants (such as PM_2.5_ and PM_10_), fuel usage, BMI, and physical activity significantly impact cognitive decline among middle-aged and elderly individuals. Additionally, drinking and marital status significantly affect cognitive decline only in the middle-aged and elderly female population.

As age increases, a decline in cognitive function is an inevitable biological process, with impacts primarily observed in neurobiology, cognitive, and behavioral psychology. Initially, aging is accompanied by significant changes in the nervous system, including reductions in brain volume, neuron count, synaptic density, and neurotransmitter levels, which directly lead to slower information processing, impaired memory, and difficulty concentrating [21, 22]. Additionally, reduced cerebral blood flow and long-term chronic ischemia further increase the risk of neurodegenerative lesions [23]. Cognitive impairment is particularly pronounced, manifesting as declines in short-term and working memory, reduced executive function, slower reaction times, and weakened verbal fluency and vocabulary retrieval speed [24]. Aging also significantly increases the incidence of neurodegenerative diseases (such as Alzheimer’s disease and vascular dementia), which, in turn, exacerbates cognitive decline [25]. Psychologically and behaviorally, older adults often exhibit reduced adaptability and diminished learning efficiency, possibly related to social isolation, loneliness, and a lack of social stimulation, further amplifying the extent of cognitive decline [26]. Moreover, environmental factors such as inadequate nutrition, lack of physical activity, sleep disorders, and long-term exposure to pollutants also accelerate brain tissue damage to some extent, posing multiple threats to cognitive function [27]. These internal and external factors collectively make aging a primary driver of cognitive decline.

Our study found that air pollution—namely ambient PM_2.5_ and indoor combustion of non-clean household fuels for heating and cooking—was the most significant contributor to cognitive decline in both men and women. PM_2.5_ induces neuronal damage via oxidative stress and neuroinflammation, disrupts the blood–brain barrier, and promotes atherosclerosis and microvascular dysfunction, leading to cerebral hypoperfusion and cognitive deterioration [28, 29]. Moreover, air pollution may indirectly exacerbate cognitive decline through behavioral factors such as emotional regulation and decision-making, which may underlie PM_2.5_’s primacy as a predictor of cognitive decline. Fine particulate matter and carbon monoxide generated by indoor combustion of non-clean fuels similarly provoke oxidative stress, chronic hypoxia, and cerebrovascular impairment, accelerating neuronal injury and cognitive impairment [30, 31]. Notably, the cognitive effects of indoor air pollution were more pronounced in women, potentially owing to women’s predominant domestic cooking roles in many sociocultural contexts, increased respiratory sensitivity to pollutants, and the modulatory influence of estrogen on oxidative stress pathways [32]. In contrast, outdoor air pollution (e.g., PM_2.5_ and PM_10_) had a greater impact on men’s cognitive function, possibly due to longer outdoor exposure and a more reactive immune response to pollutant-induced inflammation [33]. Additionally, pollution-related psychological stress and behavioral changes—such as heightened health concerns among women owing to indoor pollution and increased stress responses among men from outdoor exposures—may further widen sex disparities in cognitive decline [34, 35].

Regular physical activity significantly contributes to improved cognitive function, consistent with previous studies. Physiologically, exercise increases the expression of brain-derived neurotrophic factor (BDNF), thereby promoting neuronal growth and synaptic plasticity [36]. It also enhances cerebrovascular function by increasing cerebral blood flow and oxygen delivery, which reduces the risk of vascular injury [37]. Moreover, physical activity effectively lowers systemic and central nervous system levels of inflammatory markers—such as interleukin-6 (IL-6) and C-reactive protein (CRP)—thereby mitigating oxidative stress and inflammation–mediated neural damage [38]. Recent animal experiments further demonstrate that long-term exercise training can suppress the Toll-like receptor (TLR)–NF-κB inflammatory signaling pathway, reducing air pollution–induced neuroinflammation [39]. Psychologically, regular exercise alleviates negative affect—such as anxiety and depression—indirectly bolstering cognitive performance [40]. Therefore, encouraging middle-aged and older adults to engage in regular moderate-intensity physical activity represents an effective strategy for preventing and attenuating cognitive decline.

Interestingly, our study also demonstrated a relationship between BMI and cognitive function. Underweight individuals exhibited the most rapid cognitive decline, whereas overweight and obese participants experienced the slowest decline. Several mechanisms may underlie this phenomenon. First, adipose tissue functions as an endocrine organ by secreting anti-inflammatory cytokines—such as adiponectin and interleukin-10—which may partly attenuate neurodegeneration [41]. Second, individuals with higher BMI possess greater energy reserves, potentially conferring resilience against age- or disease-related metabolic stress [42]. Conversely, underweight status may indicate poorer nutritional status or elevated systemic inflammation, both of which could accelerate cognitive deterioration [43]. However, elevated BMI is also linked to chronic conditions—such as cardiovascular disease and type 2 diabetes—that are known to exacerbate cognitive decline [41]. Therefore, although a higher BMI may afford some short-term neuroprotective benefits, its long-term impact on cognitive health is likely complex and bidirectional. The protective association observed with overweight and obesity may be limited to a specific BMI range, whereas excessive adiposity could precipitate additional health complications, warranting further investigation in future research.

Our study revealed that, in the ranking of variable importance for cognitive function, physical activity surpassed BMI in men, whereas the opposite was true in women. This sex difference may stem from a combination of physiological and socio-behavioral factors: in women, adipose tissue secretes hormones such as estrogen and adiponectin that exert neuroprotective effects by reducing neuroinflammation, enhancing neuronal function, and promoting synaptic plasticity; consequently, BMI’s protective association with cognition is more pronounced than that of physical activity [44]. Moreover, middle-aged and older women typically engage in lower-intensity daily activities than men, which may attenuate the cognitive benefits of exercise [45]. Women are also more vulnerable to changes in nutritional status and body composition (e.g., osteoporosis, sarcopenia), so BMI may more directly reflect their overall health and metabolic reserves, thereby correlating more closely with cognitive outcomes [46]. In contrast, men generally perform higher-intensity and more regular physical activities, yielding greater improvements in cerebral blood flow, reductions in oxidative stress, and suppression of inflammation—effects that render physical activity more important than BMI for men’s cognitive health [47].

Although our study did not analyze the effects of meditation on cognitive function due to limitations of the CHARLS database, this remains an area of interest. Physical activity, as a non-pharmacological and low-cost intervention, should be promoted to delay cognitive decline; however, some older adults may be unable to maintain regular exercise because of mobility constraints, in which case meditation could serve as a more accessible alternative or complement. Meditation may benefit cognition through multiple mechanisms, including increasing gray matter density in the hippocampus and prefrontal cortex to enhance learning and memory, strengthening functional connectivity between the default mode and executive control networks, modulating cortical excitability to improve neuronal plasticity and processing speed, enhancing cerebral microcirculation and endothelial function, regulating cerebrovascular risk factors such as hypertension and hyperglycemia, lowering stress hormone levels and inhibiting inflammatory pathways to reduce neuroinflammation, protecting telomeres to delay cellular aging, and improving mood and sleep quality to indirectly support attention and cognitive performance [48–50]. A randomized controlled trial found no significant difference between mindfulness-based stress reduction and physical exercise in improving memory and executive function in older adults [51], and systematic reviews suggest that mindfulness interventions may even outperform exercise in alleviating cognitive impairment [52]. Therefore, including meditation-related variables in our study could yield importance rankings comparable to or even exceeding that of physical activity.

The study’s strengths include its use of the nationally representative CHARLS cohort, which provides a large and geographically diverse sample that enhances the generalizability of our findings. We also conducted a panoramic assessment of cognitive decline in middle-aged and older adults by integrating multidimensional factors—environmental exposures, lifestyle behaviors, and health status—and identified air pollution as a primary, modifiable environmental risk factor, thereby offering a solid empirical basis for developing gender-sensitive, personalized intervention strategies. Several limitations should be noted. First, to protect participant privacy, CHARLS only provides city-level residence data, which may result in nondifferential misclassification of individual exposure levels, introducing measurement error and residual confounding; we sought to minimize this by using high-resolution, high-quality meteorological data. Second, cognitive function was evaluated solely through standardized questionnaires and cognitive tests without incorporation of neuroimaging or biomarker indicators, limiting our ability to explore underlying neuropathological mechanisms. Third, our inclusion and exclusion criteria omitted individuals with brain injury, intellectual disability, or memory disorders; if these conditions are linked to accelerated cognitive decline, their exclusion could introduce selection bias and weaken external validity. Finally, we were unable to fully adjust for all potential confounders—such as dietary patterns, socioeconomic status, housing conditions, and number of children—which may also meaningfully affect cognitive outcomes.

## Conclusion and recommendation

In summary, our study demonstrates that age is the primary risk factor for cognitive decline, while modifiable environmental and lifestyle exposures—particularly ambient and household air pollution (PM_2.5_ and solid cooking fuels), body mass index, and physical activity—also play significant, sex-specific roles. Therefore, targeted interventions to improve air quality, promote clean fuel use, maintain healthy body weight, and encourage regular exercise may help slow cognitive deterioration among China’s aging population.

We recommend that future research proceed in the following areas: first, further validate the nonlinear relationship between body weight and cognitive function, especially whether protective effects at different weight statuses exhibit a threshold and the potential negative impact of excessive obesity, by integrating metabolic, biomarker, and neuroimaging data to explore the specific mechanisms through which adipose tissue regulates cognition; second, adopt more refined longitudinal designs with longer follow-up to dynamically assess how these factors influence cognition at different ages, and apply causal inference methods (such as randomized controlled trials or quasi-experimental designs) to further verify the pathways by which they act; third, explore in depth the biological and social mechanisms underlying sex differences—for example, whether the pronounced effects of marital status and sleep duration in women are linked to particular psychological or physiological characteristics—which will aid in developing more gender-tailored intervention strategies.

## Electronic supplementary material

Below is the link to the electronic supplementary material.

Supplementary Material 1

## Data Availability

The population data used in this study were obtained from the China Health and Retirement Longitudinal Study (CHARLS), which is publicly available at http://charls.pku.edu.cn/. The air pollution data were sourced from the Yangtze River Delta Science Data Center, National Earth System Science Data Sharing Infrastructure, National Science & Technology Infrastructure of China (http://geodata.nnu.edu.cn/). Additional datasets generated or analyzed during this study are available from the corresponding authors upon reasonable request.

## Abbreviations

BMI: Body Mass Index
CHARLS: China Health and Retirement Longitudinal Study
CHAP: China Air Pollution dataset
CO: Carbon Monoxide
IPAQ: International Physical Activity Questionnaire
MAR: Missing at Random
MET: Metabolic Equivalent of Task
NO₂: Nitrogen Dioxide
O₃: Ozone
OOB: Out-of-Bag
PA: Physical Activity
PM_2.5_: Particulate Matter with aerodynamic diameter ≤ 2.5 μm
PM_10_: Particulate Matter with aerodynamic diameter ≤ 10 μm
SO_2_: Sulfur Dioxide
TICS: Telephone Interview for Cognitive Status

## References

[1] Suwalska A, Pałys W, Łojko D, et al. Analysis of sociodemographic, clinical, and lifestyle factors associated with cognitive aging. Eur Psychiatry. 2023;66(Suppl):S172.

[2] Hong C-S, Sun L, Liu G, et al. Response of global health toward the challenges presented by population aging. China CDC Wkly. 2023;5:884–7. 37814614 10.46234/ccdcw2023.168 PMC10560387

[3] Yu J, Collinson S, Liew T, et al. The relationship between cognitive decline and all-cause mortality is modified by living alone and a small social network: a paradox of isolation. J Aging Health. 2020;32(7–8):620–30.

[4] Zhang Z, Fang E, Zhou X, et al. Social isolation, rather than loneliness, is associated with cognitive decline in older adults: the China health and retirement longitudinal study. Aging Ment Health. 2021;25(4):564–73. 10.1017/S0033291720001014 32338228

[5] Poudel G, Barnett A, Akram M, et al. Machine learning for prediction of cognitive health in adults using sociodemographic, neighborhood environmental, and lifestyle factors. Int J Environ Res Public Health. 2022;19:10977. 36078704 10.3390/ijerph191710977 PMC9517821

[6] Lövdén M, Fratiglioni L, Glymour M, et al. Education and cognitive functioning across the life span. Psychol Sci Public Interest. 2020;21:6–41. 32772803 10.1177/1529100620920576 PMC7425377

[7] Samaras D, Chen W, Hall K, et al. Comparison of sex differences in cognitive function in older adults between high- and middle-income countries and the role of education: a population-based multicohort study. Lancet Global Health. 2021;9(6):e791–9.

[8] Wang L, Wang J, Yang J, et al. Hypertension, prehypertension, and hypertension control: association with decline in cognitive performance. J Hypertens. 2019;37(6):1208–16.

[9] Huang Z, Guo Y, Ruan Y, et al. Cognitive performance declines in older adults with type 1 diabetes: results from 32 years of follow-up. Diabetes Care. 2020;43(12):3042–9.33023989

[10] Chang HH, Tseng CH, Chien LC, et al. Associations between ambient air pollution and cognitive abilities from midlife to early old age: modification by APOE genotype. Environ Res. 2020;185:109422. 10.3233/JAD-221054 PMC10827529 36970897

[11] Li F, Qian Z, Zhao J, et al. The impact of long- and short-term exposure to different ambient air pollutants on cognitive function in China. Environ Pollut. 2022;303:119077. 33667754 10.1016/j.envint.2021.106416

[12] Kim DE, Lee YH, Jeon SY, et al. Influencing factors of cognitive function in high-risk groups of dementia in one area: focused on elderly living alone. Noin Jeongsin Yihag. 2023;27(1):1–7. 10.47825/jkgp.2023.27.1.1.

[13] Drozdowska BA, Elliott E, Taylor-Rowan M, et al. Cardiovascular risk factors indirectly affect acute post-stroke cognition through stroke severity and prior cognitive impairment: a moderated mediation analysis. Alzheimer’s Res Therapy. 2020;12(1):85. 10.1186/s13195-020-00653-y. 10.1186/s13195-020-00653-y PMC7367370 32678028

[14] Zhao Y, Hu Y, Smith JP, et al. Cohort profile: the China health and retirement longitudinal study (CHARLS). Int J Epidemiol. 2014;43(1):61–8. 23243115 10.1093/ije/dys203 PMC3937970

[15] Li X, Zhang W, Zhang W, et al. Level of physical activity among middle-aged and older Chinese people: evidence from the China health and retirement longitudinal study. BMC Public Health. 2020;20(1):1682. 33172439 10.1186/s12889-020-09671-9 PMC7653852

[16] Wei J, Li Z, Lyapustin A, et al. Reconstructing 1-km-resolution high-quality PM2.5 data records from 2000 to 2018 in China: Spatiotemporal variations and policy implications. Remote Sens Environ. 2021;252:112136.

[17] Wei J, Li Z, Wang J, et al. Ground-level gaseous pollutants (NO₂, SO₂, and CO) in China: daily seamless mapping and long-term Spatiotemporal variations. Atmos Chem Phys. 2023;23:1511–32. 10.5194/acp-23-1511-2023.

[18] Wei J, Li Z, Xue W, et al. The ChinaHighPM10 dataset: generation, validation, and Spatiotemporal variations from 2015–2019 across China. Environ Int. 2021;146:106290. 33395937 10.1016/j.envint.2020.106290

[19] Wei J, Liu S, Li Z, et al. Ground-level NO₂ surveillance from space across China for high resolution using interpretable Spatiotemporally weighted artificial intelligence. Environ Sci Technol. 2022;56(14):9988–98. 35767687 10.1021/acs.est.2c03834 PMC9301922

[20] Wei J, Li Z, Li K, et al. Full-coverage mapping and Spatiotemporal variations of ground-level Ozone (O₃) pollution from 2013 to 2020 across China. Remote Sens Environ. 2022;270:112775.

[21] Chwa WJ, López OL, Kuller LH, et al. Characterization of longitudinal brain changes in a community cohort in relation to aging and cognitive impairment. Alzheimer’s Dement. 2022;18(S5):e063783. 10.1002/alz.063783.

[22] Harada CN, Natelson Love MC, Triebel KL. Normal cognitive aging. Clin Geriatr Med. 2013;29(4):737–52. 10.1016/j.cger.2013.07.002. 24094294 10.1016/j.cger.2013.07.002 PMC4015335

[23] Chen J, Rosas HD, Salat DH. Age-associated reductions in cerebral blood flow are independent from regional atrophy. NeuroImage. 2011;55(2):468–78. 10.1016/j.neuroimage.2010.12.032. 21167947 10.1016/j.neuroimage.2010.12.032 PMC3435846

[24] Salthouse TA. Shared and unique influences on age-related cognitive change. Neuropsychology. 2017;31(1):11–9. 10.1037/neu0000330. 27808539 10.1037/neu0000330 PMC5191944

[25] Livingston G, Huntley J, Sommerlad A, et al. Dementia prevention, intervention, and care: 2020 report of the lancet commission. Lancet. 2020;396(10248):413–46. 10.1016/S0140-6736(20)30367-6. 32738937 10.1016/S0140-6736(20)30367-6 PMC7392084

[26] Lara E, Martín-María N, de la Torre-Luque A, et al. Does loneliness contribute to mild cognitive impairment and dementia? A systematic review and meta-analysis of longitudinal studies. Aging Res Reviews. 2019;52:7–16. 10.1016/j.arr.2019.03.002. 10.1016/j.arr.2019.03.002 30914351

[27] Voss MW, Vivar C, Kramer AF, et al. Bridging animal and human models of exercise-induced brain plasticity. Trends Cogn Sci. 2013;17(10):525–44. 10.1016/j.tics.2013.08.001. 24029446 10.1016/j.tics.2013.08.001 PMC4565723

[28] Nassan FL, Wang C, Kelly RS, et al. Ambient PM2.5 species and ultrafine particle exposure and their differential metabolomic signatures. Environ Int. 2021;151:106447. 33639346 10.1016/j.envint.2021.106447 PMC7994935

[29] Cai L, Yang J, Cosky EE, et al. Increased cerebral microbleeds by long-term air pollution exposure in spontaneously hypertensive rats. Neurol Res. 2021;44(3):196–205. 34461819 10.1080/01616412.2021.1968705

[30] Block ML, Calderón-Garcidueñas L. Air pollution: mechanisms of neuroinflammation and CNS disease. Trends Neurosci. 2009;32(9):506–16. 10.1016/j.tins.2009.05.009. 19716187 10.1016/j.tins.2009.05.009 PMC2743793

[31] Kilian J, Kitazawa M. The emerging risk of exposure to air pollution on cognitive decline and Alzheimer’s disease– evidence from epidemiological and animal studies. Biomedical J. 2018;41(3):141–62. 10.1016/j.bj.2018.06.001. 30080655 10.1016/j.bj.2018.06.001 PMC6138768

[32] Chen YQ, Zou C, Yuan Y, et al. Indoor air pollution from solid fuel on children pneumonia in low- and middle-income countries: a systematic review and meta-analysis. Environ Sci Pollut Res. 2022;29(17):24574–88. 10.1007/s11356-021-18293-6. 10.1007/s11356-021-18293-6 35066845

[33] Calderón-Garcidueñas L, González-Maciel A, Kulesza RJ, González-González LO, Reynoso-Robles R, Mukherjee PS, et al. Air pollution, combustion and friction derived nanoparticles, and Alzheimer’s disease in urban children and young adults. Adv Alzheimer’s Disease. 2021;8:87–104. 10.3233/AIAD210008. 10.3233/JAD-190331 31256139

[34] Halder M, Kasemi N. Household air pollution as a determinant of health status: a study on older adults in Siliguri municipal corporation, India. Sci Rep. 2025;15:10048. 40122930 10.1038/s41598-025-93311-y PMC11930994

[35] Cao Z, Zhou J, Li M, Huang J, Dou D. Urbanites’ mental health undermined by air pollution. Nat Sustain. 2023;6:470–8. 10.1038/s41893-022-01032-1.

[36] Marin Bosch B, Bringard A, Logrieco MG, et al. A single session of moderate-intensity exercise influences memory, endocannabinoids and brain-derived neurotrophic factor levels in men. Sci Rep. 2021;11:14371. 10.1038/s41598-021-93708-9. 34257382 10.1038/s41598-021-93813-5 PMC8277796

[37] Yoo R-E, Kim J-H, Moon HY, et al. Long-term physical exercise facilitates putative glymphatic and meningeal lymphatic vessel flow in humans. Nat Commun. 2025;16:3360. 10.1038/s41467-025-09035-2. 40204790 10.1038/s41467-025-58726-1 PMC11982307

[38] Binabaji S, Rahimi M, Rajabi H, et al. Effects of physical training on coagulation parameters, interleukin-6, and angiotensin-converting enzyme-2 in COVID-19 survivors. Sci Rep. 2024;14:18968. 10.1038/s41598-024-76402-3. 39152162 10.1038/s41598-024-67522-8 PMC11329640

[39] De Miguel Z, Khoury N, Betley MJ, et al. Exercise plasma boosts memory and dampens brain inflammation via clusterin. Nature. 2021;600:494–9. 10.1038/s41586-021-04122-8. 34880498 10.1038/s41586-021-04183-x PMC9721468

[40] Wu J, Xu H, Wang S, et al. Regular exercise ameliorates high-fat diet-induced depressive-like behaviors by activating hippocampal neuronal autophagy and enhancing synaptic plasticity. Cell Death Dis. 2024;15:737. 10.1038/s41419-024-06512-7. 39389946 10.1038/s41419-024-07132-4 PMC11467387

[41] McGregor ER, Lasky DJ, Rippentrop OJ, et al. Reversal of neuronal Tau pathology via adiponectin receptor activation. Commun Biology. 2025;8:8. 10.1038/s42003-024-68345-6. 10.1038/s42003-024-07391-z PMC11700159 39755746

[42] Arnold M, Buyukozkan M, Doraiswamy PM, et al. Individual bioenergetic capacity as a potential source of resilience to Alzheimer’s disease. Nat Commun. 2025;16:1910. 10.1038/s41467-025-03894-2. 39994231 10.1038/s41467-025-57032-0 PMC11850607

[43] Guo DH, Yamamoto M, Hernandez CM, et al. Beige adipocytes mediate the neuroprotective and anti-inflammatory effects of subcutaneous fat in obese mice. Nat Commun. 2021;12(1):1–14. 10.1038/s41467-021-24540-8. 34330904 10.1038/s41467-021-24540-8 PMC8324783

[44] Itoh N, Meyer CE, Suen TT, et al. Estrogen receptor Β in astrocytes modulates cognitive function in mid-age female mice. Nat Commun. 2023;14:6044. 10.1038/s41467-023-40250-7. 37758709 10.1038/s41467-023-41723-7 PMC10533869

[45] Strain T, Flaxman S, Guthold R, et al. National, regional, and global trends in insufficient physical activity among adults from 2000 to 2022: a pooled analysis of 507 population-based surveys with 5·7 million participants. Lancet Global Health. 2024;12(8):e1232–43. 10.1016/S2214-109X(24)00150-5. 38942042 10.1016/S2214-109X(24)00150-5 PMC11254784

[46] Lai Y, Ramírez-Pardo I, Isern J, et al. Multimodal cell atlas of the ageing human skeletal muscle. Nature. 2024;629:154–64. 10.1038/s41586-024-04525-1. 38649488 10.1038/s41586-024-07348-6 PMC11062927

[47] Cichoń-Woźniak J, Ostapiuk-Karolczuk J, Cieślicka M, et al. Effect of two-week rest–pause on oxidative stress and inflammation in female basketball players. Sci Rep. 2024;14:14578. 10.1038/s41598-024-74444-5. 38918542 10.1038/s41598-024-65309-5 PMC11199628

[48] Haudry S, Turpin A-L, Landeau B, et al. Decoding meditation mechanisms underlying brain preservation and psycho-affective health in older expert meditators and older meditation-naive participants. Sci Rep. 2024;14:29521. 10.1038/s41598-024-22992-0. 39604423 10.1038/s41598-024-79687-3 PMC11603193

[49] Yue WL, Ng KK, Lim J. Mindfulness-based therapy improves brain functional network reconfiguration efficiency. Translational Psychiatry. 2023;13:345. 10.1038/s41398-023-02497-w. 37951943 10.1038/s41398-023-02642-9 PMC10640625

[50] Garland EL, Hanley AW, Hudak J, et al. Mindfulness-induced endogenous theta stimulation occasions self-transcendence and inhibits addictive behavior. Sci Adv. 2022;8:eabo4455. 10.1126/sciadv.abo4455. 36223472 10.1126/sciadv.abo4455 PMC9555770

[51] Lenze EJ, Voegtle M, Miller JP, et al. Effects of mindfulness training and exercise on cognitive function in older adults: a randomized clinical trial. JAMA. 2022;328(22):2218–29. 10.1001/jama.2022.19353. 36511926 10.1001/jama.2022.21680 PMC9856438

[52] Seok JW, Kim G, Kim JU. Comparative efficacy of seven non-pharmacological interventions on global cognition in older adults with and without mild cognitive impairment: a network meta-analysis of randomized controlled trials. Sci Rep. 2024;14:8402. 10.1038/s41598-024-64331-6. 38600212 10.1038/s41598-024-58232-2 PMC11006946

