# Supplementary material for: Impact of air pollution and behavioral factors on cognitive decline among middle-aged and elderly populations in China: a retrospective cohort study based on CHARLS
Source: BMC Public Health. 2025 Jul 2;25:2198. doi: 10.1186/s12889-025-23211-3 (PMC12219787; doi:10.1186/s12889-025-23211-3)
Supplement: Supplementary file 1 — Supplementary Material 1 [file 12889_2025_23211_MOESM1_ESM.docx]

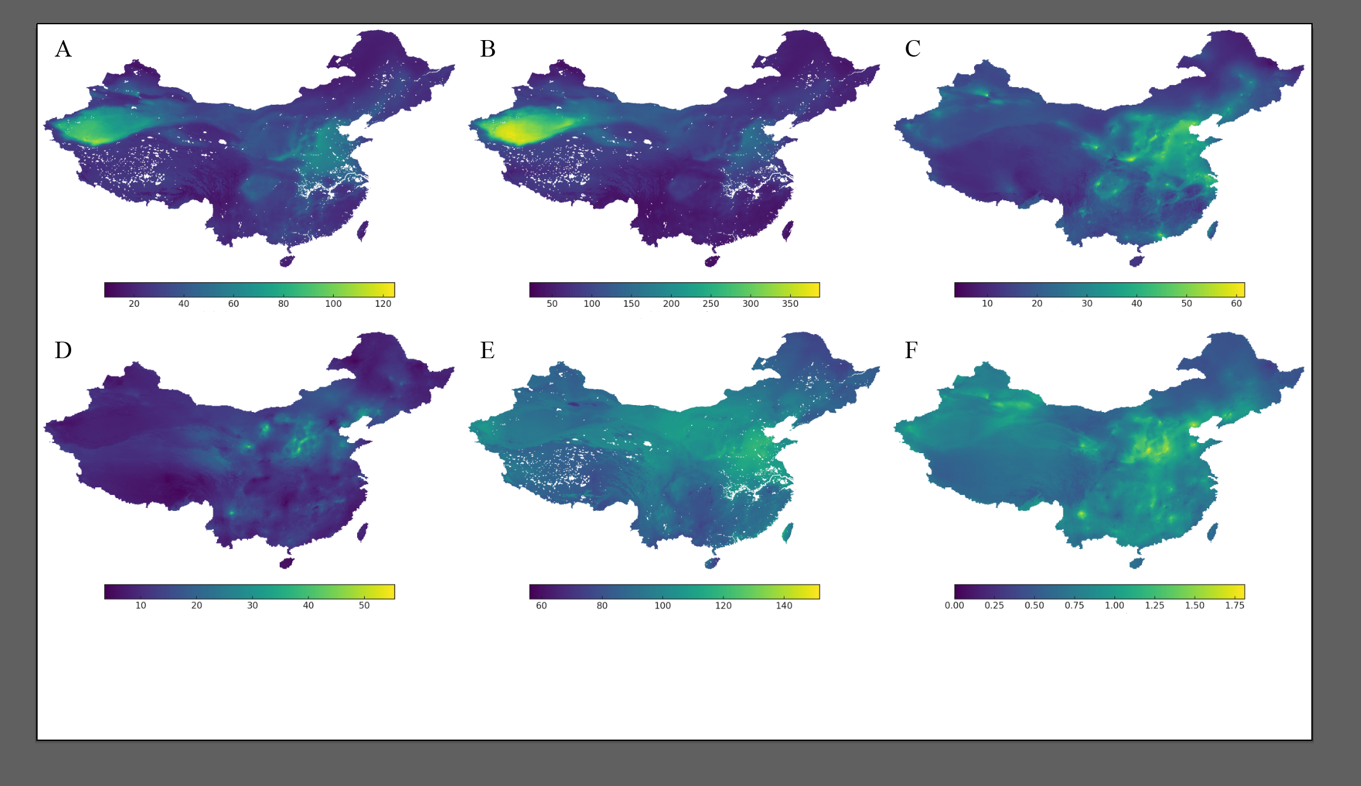


Figure S1. Annual average concentrations of air pollutants: (A) PM₂.₅; (B) PM₁₀; (C) NO₂; (D) SO₂; (E) O₃; (F) CO.

Table S1. Tenfold cross-validation, root mean square error, and mean absolute error of air pollutants

|  | PM2.5 | PM10 | NO2 | SO2 | O3 | CO |
| --- | --- | --- | --- | --- | --- | --- |
| CV-R^2^ | 0.92 | 0.90 | 0.93 | 0.84 | 0.89 | 0.80 |
| RMSE | 10.76 µg/m3 | 21.12 µg/m3 | 4.89μg/m3 | 10.07μg/m3 | 15.77 µg/m3 | 0.29 mg/m3 |
| MAE | 6.32μg/m3 | 11.22μg/m3 | 3.48μg/m3 | 4.68μg/m3 | 10.48 mg/m3 | 0.16 mg/m3 |


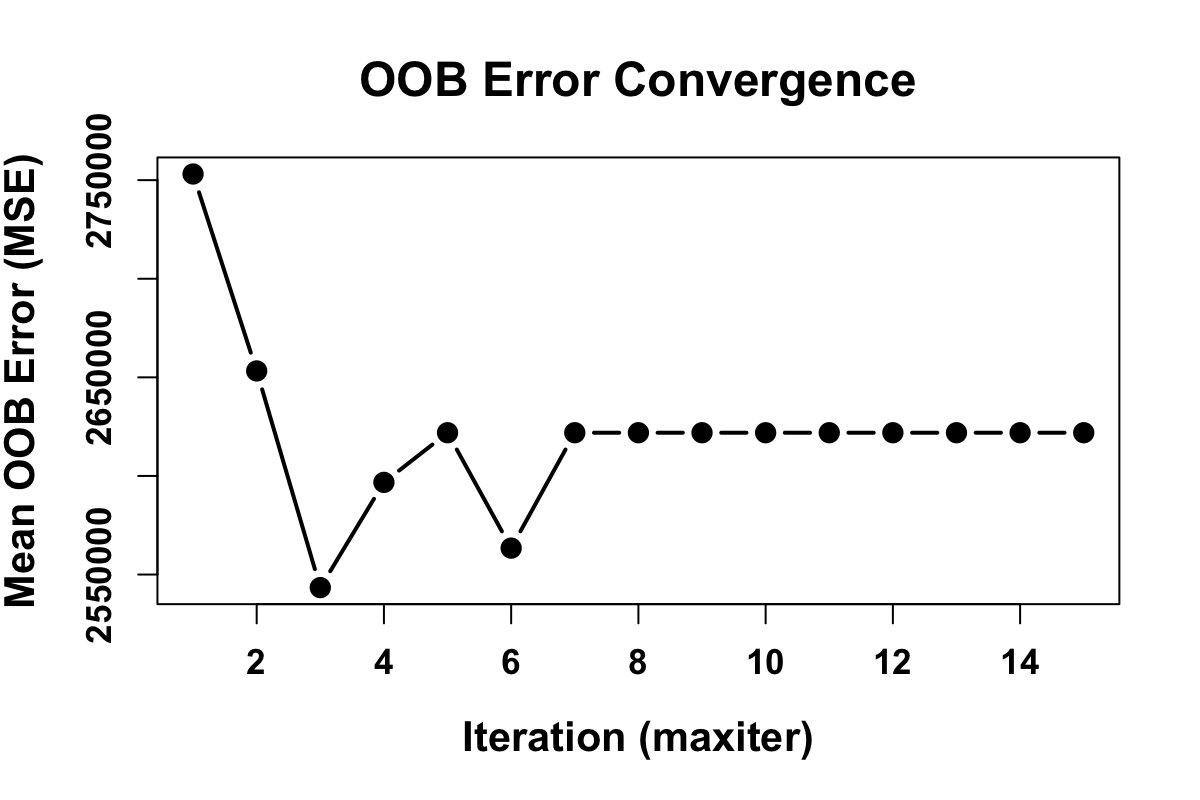


Fig S2. During random forest imputation, we found that the average out-of-bag (OOB) error stabilized at eight iterations; accordingly, we specified ntree = 100 and maxiter = 8 for the imputation, with all other parameters left at their default values.

Table S2. Before fitting the logistic regression models, we assessed multicollinearity among predictors by calculating variance inflation factors (VIF). All included variables had VIF < 5, indicating no serious multicollinearity, so no variable was excluded on that basis.

| Variables | VIF (GVIF^(1/(2·Df))) |
| --- | --- |
| PM2.5 | 3.62 |
| PM10 | 3.83 |
| NO2 | 1.33 |
| O3 | 1.16 |
| Age | 1.11 |
| SO2 | 1.72 |
| CO | 1.65 |
| Sex | 1.29 |
| Marital status | 1.04 |
| Sleep duration | 1.02 |
| Smoking | 1.17 |
| Driking | 1.13 |
| Education | 1.02 |
| Chronic disease | 1.03 |
| Physical activity level | 1.01 |

Table S3. Tests of normality and homogeneity of variance

|  | Tests of normality | | Homogeneity of variance |
| --- | --- | --- | --- |
|  | Male | Female |  |
| Age | <0.05 | <0.05 | 0.566 |
| cognition | <0.05 | <0.05 | <0.05 |
| PM2.5 | <0.05 | <0.05 | 0.344 |
| PM10 | <0.05 | <0.05 | 0.318 |
| NO2 | <0.05 | <0.05 | 0.317 |
| SO2 | <0.05 | <0.05 | 0.748 |
| O3 | <0.05 | <0.05 | 0.103 |
| CO | <0.05 | <0.05 | 0.528 |

Because the continuous variables did not satisfy tests of normality or homogeneity of variance, nonparametric tests were used.
